# Novel Algorithm for the Management of Hematospermia

**DOI:** 10.5152/tud.2020.20428

**Published:** 2020-10-23

**Authors:** Ozan Efesoy, Selahittin Çayan, Erdem Akbay

**Affiliations:** 1 Department of Urology, Mersin City Training and Research Hospital, Mersin, Turkey; 2 Department of Urology, Mersin University Faculty of Medicine, Mersin, Turkey

**Keywords:** Algorithms, hemospermia, semen

## Abstract

Hematospermia or hemospermia is traditionally defined as the presence of fresh or altered blood in semen. Several factors might cause hematospermia, including infectious, inflammatory, iatrogenic, traumatic, structural, neoplastic, vascular, and systemic factors. The main aim of evaluation is to identify significant or treatable underlying causes of hematospermia and to re-assure the patient if no causative factor is detected after full evaluation. This review aims novel management of hematospermia, including a detailed history, physical examination, appropriate laboratory investigations, and diagnostic imaging, based on underlying causes of hematospermia.

**Main Points**

- Prostate biopsy has become the single most common cause of hematospermia in the modern urological era.
- Urogenital infections are still the most common cause of hematospermia in men younger than 40 years of age.
- The main aim of evaluation is to identify significant or treatable underlying causes of hematospermia and to re-assure the patient if no causative factor is detected after full evaluation.
- The evaluation of hematospermia requires a detailed history, physical examination, and appropriate laboratory investigations and diagnostic imaging.
- First step of treatment should be allaying the anxiety of the patients and their partners via giving sufficient information about hematospermia. Proper treatment of hematospermia that include specific and empiric treatments depends on the underlying pathology.

## Introduction

Hematospermia or hemospermia is traditionally defined as the presence of fresh or altered blood in semen. It has been reported by physicians for many centuries from Hippocrates, Galen, Pare, Morgagni, Velpeau, Fournier to Guyon.^1^ Historically, majority of cases, as many as 70%, were diagnosed as functional and essential or idiopathic hematospermia. Functional causes included excessive sexual indulgence or intense sexual experiences, prolonged sexual abstinence, and interrupted coitus. Also, sudden emptying of a distended seminal vesicle causes hemorrhage “ex vacuo” was thought to be underlying cause of idiopathic hematospermia.^2^ Currently, with the use of advanced diagnostic tools, the exact cause of hematospermia can be found in most cases. There are several factors that might cause hematospermia, including infectious, inflammatory, iatrogenic, traumatic, structural, neoplastic, vascular, and systemic factors (Table 1).^3^

Hematospermia is thought to originate from the prostate gland, ejaculatory duct, seminal vesicle, vas deferens, epididymis, testis, urethra, or urinary bladder as the causative lesions in different studies.^4^ Currently, iatrogenic trauma is the most common cause of hematospermia. Because prostate biopsies have been performed more frequently in older ages, prostate biopsy has become the single most common cause of hematospermia in the modern urological era.^5,6^ However, urogenital infections are still the most common cause of hematospermia in men younger than 40 years of age.^7^ Inflammatory processes causing mucosal irritation, hyperemia, and edema of the accessory sexual glands and their ducts may lead to bleeding and the clinical manifestation of hematospermia. This inflammation can be a result of traumatic, chemical, or infectious causes.^8^ Ductal obstruction and cyst formation of accessory sexual gland and systemic factors are other causes of hematospermia. The responsible mechanism in cases with obstruction and cyst is thought to be associated with dilatation and distention, resulting in rupture of mucosal blood vessels.^9^ The most common cause of systemic factor that is associated with hematospermia is an acquired anticoagulable state secondary to drugs.^10^ Hematospermia is rarely associated with any urological malignancies, and it can be the sole symptom in men with urogenital cancer.^11^ It has been postulated that fragile aberrant vessels produced by tumor angiogenic stimuli might contribute to the situation.^12^

The exact incidence of hematospermia remains unknown and because of its self-limiting nature, most men do not observe their ejaculate, and in cases of men who notice it, some patients are hesitant to apply for medical care for this symptom.^13^ It is thought to be a relatively rare condition, and it is a symptom accounting for about 1% of urological referrals, and only a busy urologist may see more than one case per month.^2,14^ In the majority, hematospermia is a benign and self-limiting symptom. However, it is a condition that can be a source of great anxiety among patients and their partners who are often fearful of cancer or venereal disease. Most patients typically visit their primary-care physician after a single episode of hematospermia, being concerned about this serious condition.^15^

The main aim of evaluation is to identify significant or treatable underlying causes of hematospermia and to re-assure the patient if no causative factor is detected after a full evaluation.^16^ The evaluation of hematospermia requires a detailed history, physical examination, and appropriate laboratory investigations and diagnostic imaging.

## History

Detailed history is essential for evaluation of hematospermia. The features of the hematospermia, including color, amount, timing (coitus and/or masturbation), frequency, and duration, should be ascertained. It is also important to ask about relevant concomitant symptoms (Table 2).^17^“Hematospermia may be accompanied by other symptoms, but if hematospermia is the only symptom, it is defined as monosymptomatic hematospermia. Hematospermia, when recurrent, is defined as recurrent hematospermia. Also, if it continues despite the conservative or definitive treatment, it is defined as persistent hematospermia.”^10^ It is important to obtain a detailed medical history, including systemic diseases, surgical history, and the use of drugs. The patient is questioned to whether there was any external trauma in the urogenital region. Travel history to places with schistosomiasis or tuberculosis endemic areas should also be investigated.^8^

## Physical Examination

Similar to history, complete physical examination, including systemic, genital, and rectal examination, is principal for evaluation of hematospermia. The patient’s body temperature and blood pressure should be recorded, and the abdomen should be carefully examined to exclude enlargement of liver or spleen or the presence of pelvic masses. The groin, perineum, and external genitalia examination should be examined for any skin lesions, presence of hypospadias, and nodularity or induration on testis and spermatic cord. Digital rectal examination is performed to eliminate the possibility of prostate and seminal vesicle infective disorders, cysts, and pathological masses. After digital rectal examination, urethral meatus should be re-examined for presence of bloody discharge.^18,19^

## Laboratory Tests

Laboratory tests for evaluation of hematospermia include urine analysis and bacterial culture, urethral swab, Meares-Stamey four-glass test, semen analysis and culture, blood cell count, serum biochemical and coagulation parameters, and serum prostate-specific antigen levels. These tests should be tailored to individual patients.^12,18^

Urine analysis and culture-antibiogram are recommended for all patients with hematospermia, as these tests are low-cost and help confirm the presence of infection and hematuria in patients.^20,21^ However, traditionally the rate of positive culture results is low but current laboratory techniques can identify more microorganisms. The presence of hematuria is a risk factor for more serious underlying pathology. Therefore, if it occurs, a complete evaluation for hematuria should be performed.^21^

If a sterile pyuria is detected then further investigations are required to exclude chronic inflammatory disorder, such as chronic non-bacterial prostatitis, tuberculosis, schistosomiasis, and viral infections. Urethral swabs/cultures, Meares-Stamey four-glass test, semen cultures, and viral serology are tests that are used for this purpose.^16^ Urethral swab/cultures for *N. gonorrhoeae* and *C. trachomatis* should also be performed when sexually transmitted diseases are suspected or urethritis is accompanied with hematospermia.^3,16^ Semen analysis is helpful to distinguish between hematospermia and pseudo-hematospermia. It should also be performed when hematospermia is associated with low ejaculate volume and/or infertility.^22^

Hematospermia is related to an increased risk of prostate cancer. Because of this, serum prostate-specific antigen level measurement is obligatory in patients older than 40 years of age and/or when hematospermia is recurrent.^11^ Coagulation studies are recommended for patients with hematological disease, patients using any anticoagulant/antifibrinolytic drugs, and patients with recurrent hematospermia, especially >2 months, due to this situation are associated with coagulation disorders.^14^ In addition, complete blood count, creatinine and electrolytes, uric acid, and liver function tests should be performed if history and/or examination suggests chronic disorders.^12^

## Diagnostic Imaging and Endoscopy

### Vaso-Vesiculography

Vaso-vesiculography is almost never used today in the diagnostic evaluation of hematospermia. This invasive technique provides little data to detect the etiology of hematospermia. Also, it requires x-ray and may cause serious side effects, such as vasal injury and stricture.^1,23^

### Computerized Tomography

Computerized tomography has limited value in the etiologic determination of hematospermia owing to its x-ray necessity, lack of soft-tissue contrast, and limitations in the evaluation of smallcaliber structures, such as the ejaculatory duct or vas deferens and in the evaluation of the internal structure of the seminal vesicle and prostate gland.^1,24^

### Scrotal Ultrasonography

Maheshkumar et al^25^ reported persistent hematospermia as presenting symptom of testicular cancer in a patient who did not have any associated symptoms and physical examination findings. Thus, scrotal ultrasonography should be performed in patients with persistent hematospermia, or in case of any associated symptom, in order to rule out testicular pathology as an underlying cause of hematospermia.^10,25^

### Transrectal Ultrasonography

Many authors have reported that transrectal ultrasonography TRUS is a safe, simple, easily accessible, cost-effective, radiation free, and relatively noninvasive imaging modality that can clearly image seminal vesicles, ejaculatory ducts, and the prostate objectively.^4^ Its accurate diagnostic rate in patients with recurrent or persistent hematospermia is up to 95%.^26,27^ However, TRUS has some limitations, such as images obtained with ultrasonography are subject to observer variation, spatial resolution, and soft-tissue contrast may not permit complete evaluation of the ejaculatory duct and seminal vesicle, and therefore might not show the origin of bleeding, and as a result of this, it has a false positive/negative rate of approximately 50%. Thus, it has been suggested that TRUS should not be considered definitive but as primary screening modality for patients with recurrent or persistent haematospermia.^27,28^ TRUS can provide definitive diagnosis if a lesion is detected or confirmed by means of TRUS-guided seminal vesicle aspiration, prostate and seminal vesicle biopsy, and semino-vesiculography. It can also be used as a guide for treatment of hematospermia, such as TRUS-guided cyst aspiration, TRUS-guided laser incision of cyst, TRUSguided balloon dilation of ejaculatory duct in an antegrade fashion, and transurethral resection of ejaculatory duct (TURED) with TRUS-guided chromotubation.^24,29^

### Magnetic Resonance Imaging

Magnetic resonance imaging, owing to its excellent soft-tissue contrast, multiplanar capabilities, independence from the operator, and lack of ionization radiation, plays an important role as a noninvasive imaging modality in the diagnostic workup of recurrent or persistent hematospermia.^30^ Although costly, current gold standard for imaging of structural and inflammatory/infective changes in accessory sex gland and their ducts is MRI. In the presence of suspected cancer, it should include dynamic contrast imaging that provides additional information regarding tissue perfusion. In addition, MRI angiography provides further information for localizing bleeding.^24,30^ Magnetic resonance imaging has at least 80% diagnostic performance in patients with recurrent or persistent hematospermia. Endorectal phased-array coil is superior to external phased-array coil for evaluation of the prostatic region, but it causes discomfort to the patient. However, Sosna et al^31^ showed the image quality at the external coil at 3.0 T to be comparable with the endorectal coil at 1.5 T.

In a large series of patients with recurrent or persistent hematospermia, Li et al^28^ reported that there is no significant difference in the positive rate of abnormal imaging between MRI and TRUS (86.3% vs. 84.3%, *P* >.05), while MRI provides more precise causative information, particularly regarding ejaculatory duct obstruction and hemorrhage location, than TRUS. According to American College of Radiology, MRI is indicated when TRUS results are negative or inconclusive (Tables 3 and 4).^24^

### Pelvic Angiography

In the literature, pelvic angiography has been rarely reported to be useful for diagnosis of patients with persistent massive hematospermia and hematuria due to vascular masses, such as prostatic hemangioma, arteriovenous malformation, and varices. If an arterial source of hemorrhage is identified, transcatheter arterial embolization or electrofulguration can be performed during the same session as well.^32^

### Urethrocystoscopy

When hematospermia is accompanied by hematuria in elderly patients or all attempts to diagnose the disease fails, in high risk patients with recurrent or persistent hematospermia, rigid or flexible urethrocystoscopy should be considered. It allows direct vision of urethra, prostate, bladder neck, and bladder and their pathologic conditions. Concurrent massage of the prostate and seminal vesicles may be useful for localizing bleeding. Due to its very poor diagnostic performance, urethrocystoscopy is not recommended routinely for diagnostic workup of hematospermia, as well as renal tract ultrasound and intravenous urography.^33^

### Transurethral Seminal Vesiculoscopy

Transurethral seminal vesiculoscopy (TSV) is another endoscopic technique that can directly visualize etiological lesions in the ejaculatory duct and seminal vesicles with high sensitivity and specificity. A definite diagnosis rate of 93.1% was reported in the first large-scale report of patients with recurrent or persistent hematospermia.^34^ In a prospective study, Xing et al^26^ compared TSV and TRUS for the diagnosis of persistent hematospermia. They reported that overall diagnostic yield of TSV was significantly superior to that of TRUS (74.5% vs. 45.3%, *P* < .001) and the diagnostic yield of combining TSV and TRUS was significantly higher than that of each modality alone (both *P* < .001). In addition, TSV is not only a diagnostic but also a treatment tool for ejaculatory duct and seminal vesicle pathologies. However, even though it is a minimally invasive procedure that requires anesthesia, TSV is currently not standardized, failures still occur, long-term safety is uncertain, and can lead to complications, such as epididymitis, ejaculation abnormality, and injury of the seminal vesicle and rectum.^23,34^

### Treatment

First step of treatment should be allaying the anxiety of the patients and their partners via giving sufficient information about hematospermia. Due to natural history of hematospermia, reassurance and follow-up without any treatment will be adequate for many patients. In a prospective study investigating natural history of hematospermia in patients who underwent watchful waiting without any empirical treatment, Furuya et al^9^ reported that the persistence rates of hematospermia were 57.7% at 1 month, 34.2% at 3 months, 23.3% at 6 months, 12.5% at 1 year, and 7.6% at 2 years. Iatrogenic causes of hematospermia usually resolve spontaneously within approximately 10 ejaculations or on an average 11 days, but it can continue for up to 2 months.^5,6^

Proper treatment of hematospermia that include specific and empiric treatments depends on the underlying pathology. If urogenital infection is affirmed, the treatment of appropriate antibiotic, antiparasitic, or antiviral agents is indicated on the basis of the sensitivity of the cultured organism.^20^ Also, systemic conditions, if any, should be treated appropriately.

If infection is suspected, but no pathological findings are determined, empiric treatment is suitable. Antibiotics should be capable of penetrating the prostate-blood barrier (e.g., fluoroquinolones, tetracyclines, macrolides, trimethoprim-sulfamethoxazole, and metronidazole) and non-steroidal anti-inflammatory drugs (e.g., ibuprofen and celocoxib). In a large, but non-controlled and retrospective study, Zargooshi et al.^15^ reported that in 94.9% of patients, hematospermia did not recur after empirical treatment (ciprofloxacin plus celocoxib). Other empiric treatments contain antifibrinolytic, antiandrogenic, and estrogenic agents, such as aminocaproic acid,^35^ finasteride,^36^ and ethinyl estradiol.^37^ These agents should only be used in selected patients with persistent hematospermia, as there is insufficient evidence on the use of such agents.

Specific surgical treatment is given, if it is required, when vascular anomalies, polyps, calcification, cysts, or other pathological conditions are detected. Urethroscope-guided electroexcision and/or fulguration of polyps and vascular anormalies in posterior urethra,^29^ direct drug injection to seminal vesicles guided by TRUS,^38^ TRUS-guided cyst aspiration with or without sclerotherapy,^39^ TRUS-guided transurethral laser incision of cyst,^40^ transurethral unroofing or laparoscopic management of seminal vesicle cysts,^41^ TURED with or without TRUS-guided choromotubation,^42^ TRUS and fluoroscopic assisted transurethral incision of ejaculatory ducts,^43^ urethroscopy and TRUS or CT/MRI-guided recanalization and dilatation of ejaculatory duct,^22,44^ TSV-guided incision of obstructed ejaculatory duct, coagulate hemorrhagic mucosa, and fragment stones in the ejaculatory duct or seminal vesicle by using electric or laser energy systems^7,45^ are methods that can be performed for this purpose.

**Figure 1.**
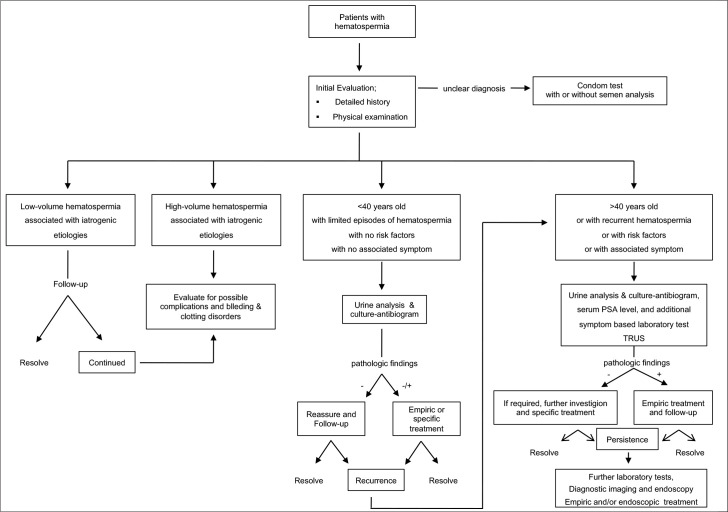
Novel Algorithm for the Management of Hematospermia.

In conclusion, to address how the novel algorithm in the management of hematospermia should be, the first step is to rule out pseudo-hematospermia, which could be because of bleeding from sexual partner source, hematuria, urethral bleeding, and melanospermia.^19^ If in doubt, a “condom test” should be performed, where the semen is collected and then checked for blood.^20^ Once true hematospermia has been affirmed, the diagnostic process should be initiated with a clinical history and physical examination. Afterward, 3 key factors help guide further evaluation (Figure 1): age, duration of hematospermia, and presence of concomitant symptoms or risk factors since recurrent or persistent hemospermia may indicate a more serious underlying pathology, especially in patients over 40 years of age.^17^ However, the evidence basis for the investigation and management of haematospermia remains lacking.^13,33^

## Figures and Tables

**Table 1.** Etiology of Hematospermia, Modified From Suh et al.^3^

Inflammation and Infections	Iatrogenic/Trauma
Hemorrhage ex vacuo	Prostate biopsy
Calculi of seminal vesicles, ejaculatory duct, prostate, urethra, bladder, or ureter	Prostatic injection, brachytherapy, cryotherapy, high-intensity focused ultrasound therapy
Prostatitis, seminal vesiculitis, urethritis, epididymo-orchitis	Pelvic external beam radiotherapy
*Viral*; HSV, CMV, HPV, Zika virus, HIV	External trauma to perineum genitalia or pelvis
*Bacterial; C. trachomatis, N. gonorrhoeae, T. pallidum*,	Coital or auto-erotic trauma
*M. hominis, U. urealyticum, E. feacalis*, etc.	Urethral catheterization/self-instrumentation
*Parasitic; S. haematobium, E. granulosus*	Ureteral/Urethral stents
Tumors	Vasectomy
*Benign*	Orchiectomy
Granulation, papillary adenoma, adenomatous polyps	Hemorrhoidal sclerotherapy
Condylomata accuminata	Drugs (aspirin, anticoagulants, and panitumumab)
Leiomyoma and adenomyosis of seminal vesicles	Ductal obstruction and cysts of accessory sexual glands
Tumors of spermatic cord and prostatic utricle	Ejaculatory duct obstruction
Angioleiomyoma of the testicle	Mullerian duct, prostatic, utricular, ejaculatory duct cysts
*Malignant*	Dilatation of seminal vesicles
Carcinoma of the prostate, testis, and seminal vesicle	Cyst and diverticula of seminal vesicles
Sarcoma of prostate and seminal vesicles	Urethral stricture
Intraductal carcinoma	Benign prostatic obstruction
Metastatic disease such as melanoma	Systemic factors
Rare tumors	Severe systemic hypertension
Vascular abnormalities	Cirrhosis of the liver, hyperuricemia
Abnormal veins in the prostatic urethra, prostatic telangiectasia	Amyloidosis of seminal vesicles
Hemangioma of urethra and spermatic cord	Scurvy
Arteriovenous/vascular malformation Semino-vesico-venous and vaso-venous fistulas	Hematological disorders (Hemophilia, von Willebrand disease, leukemia, and lymphoma)

**Table 2.** Symptoms That Accompany Hematospermia and Possible Causes

Symptom	Underlying Pathology
Urethral discharge	Urethritis, prostatitis
Dysuria	Cystitis, urethritis, prostatitis
Hematuria	Cystitis, urethritis, prostatitis, prostatic or urethral polyps, bladder or prostate cancer
Lower urinary tract symptoms	Cystitis, urethritis, prostatitis, primary or secondary involvement of the bladder or bladder outlet
Pelvic/perineal pain/discomfort	Cystitis, urethritis, prostatitis, obstruction of ejaculatory duct
Painful ejaculation - Orgasmalgia	Prostatitis, obstruction of ejaculatory duct
Low ejaculate volume, infertility	Obstruction of ejaculatory duct
Testicular pain and swelling	Epididymal or testicular infections or tumor
Systemic symptoms (weight loss, night sweats, chills, fewer, bone pain, etc.)	Infectious diseases, genitourinary cancer
Overt mucosal bleeding, bleeding into the skin	Bleeding and clotting disorders, leukemia, lymphoma, drugs

**Table 3.** ACR Appropriateness Criteria^®^ on Hematospermia; Patients Under 40 Years of Age with Transient and Monosymptomatic Hematospermia (Modified from Expert Panel on Urologic Imaging, 2017).^24^

Radiologic Procedure	Appropriateness Rating	Relative Radiation Level	Effective Dose Estimate Range(mSv)
Transrectal ultrasound	3	0	0
MRI pelvis			
without iv contrast	3	0	0
without and with iv contrast	3	0	0
CT pelvis			
without iv contrast	1	3	1–10
with iv contrast	1	3	1–10
without and with iv contrast	1	4	10–30
Arteriographypelvis	1	4	10–30

Note: ACR: American College of Radiology; MRI: magnetic resonance imaging; CT: computerized tomography; iv: intravenous.

Rating scale: 1, 2, 3 = usually not appropriate; 4, 5, 6 = may be appropriate; 7, 8, 9 = usually appropriate.

**Table 4.** ACR Appropriateness Criteria^®^ on Hematospermia; Patients Over 40 Years of Age, or Any Age with Recurrent Hematospermia, or Hematospermia Accompanied by Associated Symptoms or Signs of Disease (Modified From Expert Panel on Urologic Imaging, 2017).^24^

Radiologic Procedure	Appropriateness Rating	Relative Radiation Level	Effective Dose Estimate Range(mSv)
Transrectal ultrasound	8	0	0
MRI pelvis			
‰without iv contrast	7	0	0
‰without and with iv contrast	8	0	0
CT pelvis			
‰without iv contrast	1	3	1–10
‰with iv contrast	2	3	1–10
‰without and with iv contrast	1	4	10–30
‰Arteriography pelvis	2	4	10–30

ACR, American College of Radiology; CT, computerized tomography; iv, intravenous; MRI, magnetic resonance imaging.

Rating scale: 1, 2, 3 = usually not appropriate; 4, 5, 6 = may be appropriate; 7, 8, 9 = usually appropriate.
